# Effective Reduction of Volumetric Thermal Expansion of Aromatic Polyimide Films by Incorporating Interchain Crosslinking

**DOI:** 10.3390/polym10070761

**Published:** 2018-07-11

**Authors:** Shinji Ando, Mari Harada, Tomohiro Okada, Ryohei Ishige

**Affiliations:** Department of Chemical Science and Engineering, Tokyo Institute of Technology, Ookayama 2-12-1-E4-5, Meguro-ku, Tokyo 152-8552, Japan; mharada@polymer.titech.ac.jp (M.H.); tokada_polymer@yahoo.co.jp (T.O.); rishige@polymer.titech.ac.jp (R.I.)

**Keywords:** volumetric thermal expansion, polyimide, crosslink, chain packing, isomerism, anisotropy, local motion

## Abstract

To develop a facile method for reducing the coefficient of volumetric thermal expansion (CVE) of polymer films, the thermal expansion behaviors of thermally cross-linkable polyimide (PI) films with isomeric diamine structures were investigated via thermal mechanical analyses and optical interferometry measurements. The degree of crosslinking of the PI films containing the diphenylethynylene (Ph–C≡C–Ph) structure in the main chain was characterized by far-infrared (far-IR) spectra and density functional theory (DFT) calculations, and variations in the CVE induced by thermal crosslinking were quantitatively estimated. The crosslinking reactions effectively reduced the CVEs of the PI films by suppressing intermolecular free volume expansion and local molecular motions promoted at elevated temperatures. The lowest CVE value observed for a crosslinked PI cured at 400 °C (+98 ppm/K at 80–280 °C) was one of the smallest values reported to date in polymers. Incorporating interchain crosslinking into the main chain is an effective method for reducing the CVE of aromatic polymers.

## 1. Introduction

Recently, the dimensional stability of polymeric dielectric layers in electronic devices as well as packaging against repeated thermal cycles during fabrication have become increasingly important with rising demand for higher-density integrated devices. Fully aromatic polyimides (PIs) have been used in the electric, electronic, and aerospace industries because of their high thermal stability, chemical and radiation resistance, flame retardancy, mechanical strength, good flexibility, and excellent dielectric properties. The high degree of ductility and inherently low thermal expansion of PI films are beneficial for a variety of microelectronic applications [1,2,3].

The relationship between the chemical structure of aromatic PIs and their anisotropic linear and volumetric thermal expansion (VTE) behaviors has been recently characterized [4,5,6]. PIs with bulky side groups, such as –CH_3_ and –CF_3_, or those with flexible linkages, such as –O– and –CO–, in the main chain exhibit larger coefficients of volumetric thermal expansion (CVE) compared to PIs containing rigid and linear main chain structures without side groups [4]. In addition, poly(*p*-phenylene-3,3′,4,4′-diphenyldiimide) (*s*BPDA/PPD) PI films with a rigid molecular structure and thermally imidized under very fast/slow heating rates exhibited different CVEs, owing to their varying degrees of main chain packing [5]. Moreover, films with dense molecular packing exhibited much smaller CVEs than those with loose molecular packing. This indicates that the control of chain packing via preparation conditions is key for reducing the VTE of PIs.

Introducing network structures with interchain crosslinking is effective for controlling aggregation and polymer density. For PIs, two thermally cross-linkable groups—ethynyl (–C≡CH) and phenylethynyl (–C≡C–Ph)—at the termini of the main chains are widely used to enhance thermal and chemical stability [7,8,9]. However, Liou et al. [10] report the VTE behavior of PIs as end-capped with the thermally cross-linkable groups of bismaleimide or 4-phenylethynylphthalic anhydride, in which the promotion of crosslinking induces a lowering of density and an increase in CVE. This clearly indicates that the incorporation of crosslinked structures at the chain termini does not induce densification. In contrast, we analyzed the crosslinked structure of a PI derived from 3,3’-diaminodiphenylacetylene, using ^13^C cross polarization/magic angle spinning (CP/MAS) NMR, and determined that the most probable crosslink mechanism is Diels-Alder cycloaddition between two phenylethynyl groups, which affords polycyclic aromatic structures containing biphenyl linkages [11]. Moreover, benzophenone (Ph–CO–Ph) incorporated into the main chains is a photo-cross-linkable group, in which the excited triplet state of benzophenone undergoes hydrogen abstraction with a spatially adjacent benzyl-positioned hydrogen, and successively forms a crosslinked structure with –OH groups [12].

Despite there being many studies about main chain polymer crosslinking, the VTE behavior of PIs undergoing crosslinking reactions at diphenylethynylene (Ph–C≡C–Ph) or benzophenone moieties has not been reported. In this study, the effects of thermal crosslinking between main chains in both the anisotropic linear and VTE behaviors were investigated for fully aromatic PIs with a diphenylethynylene moiety in the main chain.

## 2. Experimental

### 2.1. Sample Preparation

**Scheme 1:**
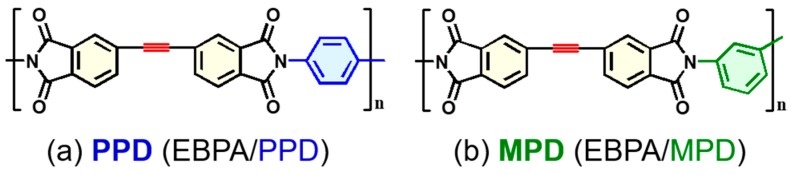
Chemical structures of polyimides (PIs) containing diphenylethynylene structure: (**a**) *p*-phenylenediamine (PPD) (4,4′-(ethyne-1,2-diyl)diphthalic dianhydride (EBPA)/PPD) and (**b**) *m*-phenylenediamine (MPD) (EBPA/MPD).

First, 4,4′-(ethyne-1,2-diyl)diphthalic dianhydride (EBPA, Tokyo Kasei Kogyo Co., Ltd., Tokyo, Japan) was dried at 170 °C for 6 h in vacuo. Subsequently, *p*-phenylenediamine (PPD) and *m*-phenylenediamine (MPD), purchased from Tokyo Kasei Kogyo Co., Ltd., were purified by sublimation under reduced pressure. The precursors of poly(amic acid)s (PAAs) were polymerized from equimolar amounts of dianhydride and diamine dissolved in *N*,*N*-dimethylacetamide (DMAc), which were prepared under a dry nitrogen atmosphere. The PAA solutions were spin-coated onto a silicon substrate, followed by baking under several conditions for thermal imidization to obtain PI films. The chemical structures of the PIs used in this study are shown in Scheme 1, and their thermal curing conditions are presented in Table 1. Hereafter, EBPA/PPD PIs are called PPD-PIs, and EBPA/MPD PIs are called MPD-PIs. The number after the sample name represents the final curing temperatures in °C. All curing procedures were conducted under a dry nitrogen flow. The thicknesses of the resultant PI films were controlled, and ranged from 11 to 13 μm for the precise measurement of the coefficient of thermal expansion (CTE) and CVE.

### 2.2. Measurements

UV/visible absorption spectra were measured using a JASCO V-670 (Jasco Corp., Tokyo, Japan) spectrophotometer from 300 to 800 nm at a 2 nm resolution. All far-IR absorption spectra were obtained using a FT/IR-6100 spectrometer (Jasco Corp) at room temperature in a vacuum chamber. Far-IR spectra were collected with 150 scans, at a resolution of 2 cm^−1^, from 650 to 100 cm^−1^. Transmission far-IR spectra of the PI films were collected using a *p*-polarized incident beam at the Brewster’s angle (~58°), in order to suppress interference caused by surface-reflection. A PL-82 wire-grid polarizer (Jasco Corp) was attached close to the PE window of the spectrometer.

A robust single-reflection accessory (JASCO ATR-Pro-One) with a germanium internal reflection element (IRE, *n* = 4.0, incidence angle = 45.0°) was used for the attenuated total reflection (ATR)-mid-FT-IR spectra measurements. The sample film was pressed to contact the IRE, using a stainless steel anvil with a special mechanism that provided a preset pressure. A silicon wafer and a 1-mm-thick silicone rubber sheet were placed between the sample and the anvil to ensure uniform pressure at the contact position. The ATR-mid-FT-IR spectra were recorded using a FT/IR-4200 spectrometer (Jasco Corp) , with 50 scans per spectrum at a 4 cm^−1^ resolution.

In-plane (*n*_TE_) and out-of-plane (*n*_TM_) refractive indices of the PI films were measured using a prism coupler (PC-2010, Metricon Corp., Pennington, NJ, United States) at a wavelength of 1310 nm at room temperature. The average refractive index, *n*_av_, was calculated using the relation *n*_av_^2^ = (2*n*_TE_^2^ + *n*_TM_^2^)/3. The in-plane/out-of-plane birefringence (∆*n*) of the PI films was calculated as *n*_TE_ − *n*_TM_.

Transmission wide-angle X-ray scattering (WAXS) measurements of the PI films with a synchrotron X-ray source were performed at the Photon Factory in High Energy Accelerator Research Organization, Tsukuba, Japan, at the BL-10C beamline. The two-dimensional (2D) WAXS pattern was collected using a 2D detector (PILATUS3-300 KW, Dectris Ltd., Baden-Daettwil, Switzerland), which had a resolution of 1475 × 1679 pixels with 172 × 172 μm^2^ in each pixel dimension, and an exposure time of 180 s. The wavelength λ of the X-ray was set to 0.89 Å. The 2D WAXS pattern was azimuthally averaged to obtain one-dimensional (1D) scattering intensity profiles, and the intensity data were plotted with respect to the magnitude of the scattering vector *q* defined as follows.

(1) $$q=\frac{4\pi \cdot \sin\theta }{\lambda }$$

where θ and λ represent the half value of the scattering angle and the wavelength of the incident X-ray, respectively. Thermomechanical analysis (TMA) was conducted using TMA-60 (Shimadzu Corp., Kyoto, Japan) with a fixed load of 3.0 g and a heating rate of 2 °C/min under a nitrogen flow. The film size for the TMA was 5 mm wide and 20 mm long. After preliminary heating to 150 °C (first heating) and successive cooling to room temperature in the TMA chamber, the data were collected during the second heating run to eliminate the effects of the absorbed water. The coefficient of linear thermal expansion (CTE) in the in-plane direction of the PI film, CTE_//_, was estimated from the variation in length according to the following equation:

(2) $${\mathrm{CTE}}_{//}=\frac{1}{l_{//}\left(T_{0}\right)}\cdot \frac{dl_{//}}{dT}$$

where *T*, *T*_0_, and *l*_//_ represent the absolute temperature, reference temperature (60 °C), and length of the film, respectively.

Temperature variation over the thickness of the PI films was precisely measured using optical interferometry during the heating process [5,6]. The transmission spectra in the near-IR region were used, owing to the absence of absorptions. All near-IR transmission spectra were collected between 7200 and 1500 cm^−1^ using a FT/IR-4200 spectrometer (Jasco Corp) at a resolution of 4 cm^−1^, with 16 scans under a nitrogen flow. An automatic heating stage (10036L, Linkam Sci. Instr., Tadworth, UK) with a transmission hole of 2 mm-φ and BaF_2_ windows was placed in the FT-IR chamber, and the near-IR spectra from 7200 and 1500 cm^−1^ were obtained at preset temperatures. The background (air) spectra were measured at every preset temperature, followed by sample measurements. To eliminate the influence of the second harmonic absorption of C–H stretching and water vapor, the near-IR spectra from 6700 to 4500 cm^−1^, except for 6150 to 5900 cm^−1^ and 5400 to 5100 cm^−1^, were collected, in order to analyze the refractive index and film thickness of the PI films. The detailed procedures for this analysis have been reported elsewhere. The average CTE in the thickness direction (i.e., out-of-plane direction), CTE_⊥_, was estimated from thickness variation according to the equation:

(3) $${\mathrm{CTE}}_{⊥}=\frac{1}{l_{⊥}\left(T_{0}\right)}\cdot \frac{dl_{⊥}}{dT}$$

where *l*_⊥_ indicates the thickness of the PI film. The coefficient of volumetric thermal expansion (CVE) was calculated using the following equation:

(4) $$\mathrm{CVE}=\frac{1}{V\left(T_{0}\right)}\cdot \frac{dV}{dT}=2\left(1+\Delta T\cdot {\mathrm{CTE}}_{//}\right)\cdot \left(1+\Delta T\cdot {\mathrm{CTE}}_{⊥}\right)\cdot {\mathrm{CTE}}_{//}+{\left(1+\Delta T\cdot {\mathrm{CTE}}_{//}\right)}^{2}\cdot {\mathrm{CTE}}_{⊥}$$

where *V* is the volume of the PI film, and Δ*T* is defined as *T* − *T*_0_.

The tanδ curves of the PI films were obtained from the storage modulus (*E*′) and loss modulus (*E*″) by dynamic mechanical analysis (DMA). The data were corrected using a DMA-7100 (Hitachi High-Technologies Corp., Tokyo, Japan) instrument, at a heating rate of 4 °C/min. The measurements were conducted with a sinusoidal load and frequency of 10 Hz under a nitrogen flow.

### 2.3. Quantum Chemical Calculations

Density functional theory (DFT) with a three-parameter Becke-style hybrid functional (B3LYP) was adopted for calculating the optimized structures and linear polarizability of the PI films [13]. The 6-311G (d, p) basis set was used for geometry optimization, and the 6-311G+(2d, p) basis set was utilized for polarizability calculations, as well as mid- and far-IR vibrations. The scaling factor for vibrational frequencies was set to 0.96. All calculations were performed with the Gaussian-09 D.02 program package installed at the Global Scientific Information and Computing Center (GSIC), Tokyo Institute of Technology.

Herman’s orientation function of the polymer main chains along the in-plane direction (*P*_200_) can be expressed as follows [14]:

(5) $$P_{200}=\frac{3\langle \cos^{2}\theta_{i}\rangle -1}{2}$$

where θ*_i_* is the angle between the polymer chain and perpendicular direction of film. The birefringence, Δ*n*_i_, which is defined as *n*_TM_ − *n*_TE_, can be expressed as follows, using *P*_200_:

(6) $$\Delta n_{i}=P_{200}\cdot \Delta n^{0}$$

where Δ*n*^0^ is the intrinsic birefringence estimated by DFT calculations, as reported by Terui et al. [13,15] *P*_200_ values were thus estimated according to Equation (6).

The coefficient of molecular packing, *K*_p_, was estimated according to the following equation:

(7) $$K_{p}=\frac{3}{4\pi }\frac{V_{\mathrm{vdw}}}{\alpha_{\mathrm{av}}}\frac{n_{\mathrm{av}}{}^{2}-1}{n_{\mathrm{av}}{}^{2}+2}$$

where *V*_vdw_ and α_av_ are the van der Waals volume and average linear polarizability, respectively. The procedures for calculating the values of *P*_200_ and *K*_p_ of the PI films have been described in detail elsewhere [13,15].

## 3. Results and Discussion

### 3.1. Estimation of Degrees of Thermal Crosslinking Reactions

**Figure 1:**
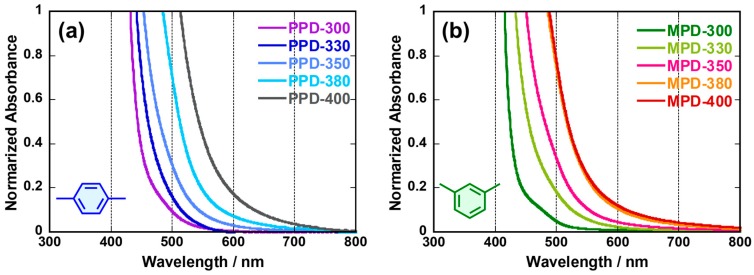
UV-Vis absorption spectra of PI films derived from (**a**) PPD and (**b**) MPD.

**Figure 2:**
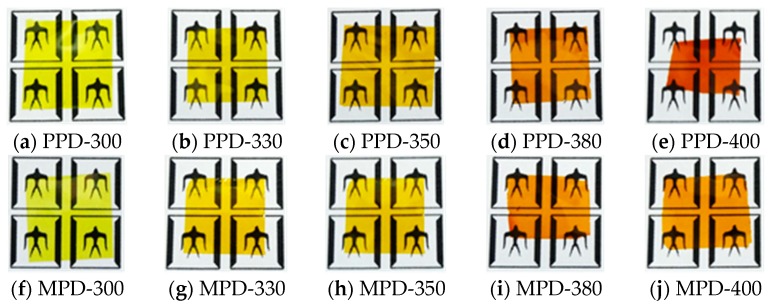
Photo images of the PI films cured at different temperatures: (**a**–**e**) PI films derived from PPD (PPD-PIs), and (**f**–**j**) PI films derived from MPD (MPD-PIs).

The UV-vis absorption spectra of the PI films are shown in Figure 1. The absorption edges of the thermally cross-linkable PI films exhibited significant bathochromic shifts with increasing final curing temperature. This indicates that the diphenylethynylene groups in the PI films cured at ˃300 °C reacted with each other to a large extent, and formed intermolecular cross-linked structures. The appearance of the PI films changed from bright yellow to dark brown, as shown in Figure 2.

**Figure 3:**
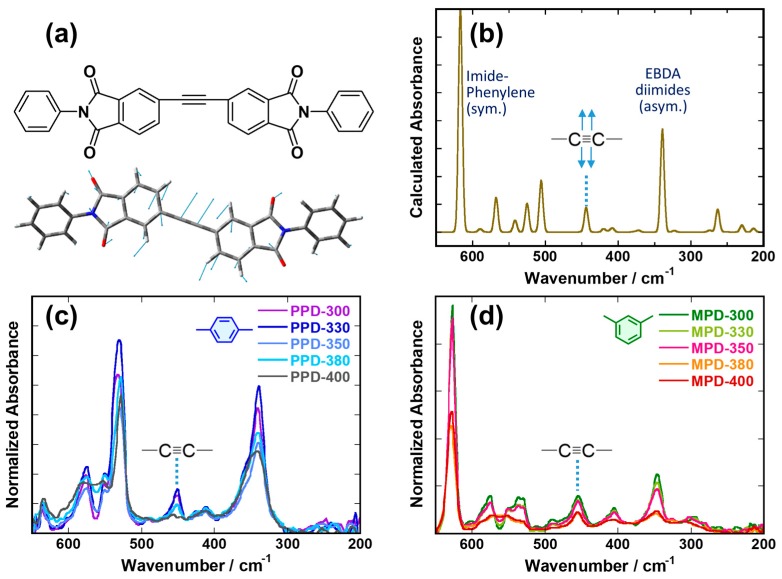
(**a**) Structure of a model used for DFT calculations, and the vibration mode corresponding to the symmetric rocking of the –C≡C– structure (445 cm^−1^); (**b**) calculated far-IR absorption spectrum of the model; and experimental far-IR absorption spectra of (**c**) PPD-PI and (**d**) MPD-PI films cured at different temperatures.

Fang et al. [16] reported that the degree of reaction at the phenylethynyl group can be detected by monitoring the IR absorption band for –C≡C– stretching at 2214 cm^−1^. However, the PIs derived from EBPA dianhydride did not exhibit –C≡C– stretching bands (Supplementary Materials Figure S1) because of the EBPA dianhydride’s IR-inactive symmetric structure. We recently reported that the far-IR spectrum of an imide model compound could be accurately reproduced using DFT calculations from 650 to 100 cm^−1^, by considering the local molecular conformations and effects of neighboring molecules [17]. In this study, DFT calculations of model compounds for EBPA predicted that the IR-active absorption modes related to the diphenylethynylene group would appear in the far-IR region at ˂500 cm^−1^. Figure 3 shows the model compound used for the DFT calculation, calculated vibrational modes, and experimental far-IR absorption spectra of the PI films cured at different temperatures. In Figure 3b–d, the calculated far-IR spectra showed good agreement with the experimental spectra of the PPD and MPD-PI films, where the calculated absorption band at 445 cm^−1^ was assigned to symmetric rocking of the –C≡C– structure. The calculated far-IR absorption spectra and the vibration modes of some representative absorption peaks of the model depicted in Figure 3a, as well as those for PPD-PIs and MPD-PIs, are displayed in Figures S2–S5 (Supplementary Materials).

To evaluate the degree of thermal crosslinking (*D*_C_) of the diphenylethynylene moiety, integral intensities of the 450 cm^−1^ absorption bands in the far-IR spectra were quantified by fitting, using a Lorentzian broadening function. As listed in Table 2, the *D*_C_ value increased with increasing curing temperature, and reached slightly higher than 40% after heating at 400 °C for 90 min. In our previous study on the curing of PI films with phenylethynyl moieties at the end of the main chain, thermal crosslinking gradually proceeded and was nearly complete (~100%) by heating at ˃350 °C [11]. In this study, the *D*_C_ value reached only ~45% after curing at 400 °C for 90 min, owing to the rigidity of the main chains and internal diphenylethynylene structure. The vigorous molecular motion required for interchain crosslinking reactions was effectively suppressed. However, the *D*_C_ of approximately 42%–45% should be sufficiently high to fix and immobilize the PI main chains in the solid films. The suppression of local molecular motion, as represented by increased β- and γ-relaxation temperatures, is important to reduce the CVEs of polymers [5,6].

### 3.2. Aggregation Structure and Molecular Chain Orientation of Polyimide Films

The average refractive indices (*n*_av_), in-plane/out-of-plane birefringence (∆*n*), orientation functions (*P*_200_), and coefficients of molecular packing (*K*_p_) of the pristine and thermally cured PI films are listed in Table 3. To calculate the *K*_p_ values of the PI films containing crosslinked structures, three model compounds were used (Supplementary Materials Scheme S1), where Model A represents the structure before crosslinking, and Models B and C represent the structures after crosslinking, which include a fused phenylnaphthalene and 1,2-diphenylbenzene, respectively. These structures were designed based on our previous study [11]. The calculated average polarizability and principal values of the polarizability tensor of Models A to C are listed in Table S1 (Supplementary Materials), along with their van der Waals volumes, intrinsic refractive indices, and birefringence. The reduced polarizability ($\bar{\alpha }$) of a repeating unit of PI at a certain degree of crosslinking (*D*_c_) was estimated, using the following formula.

(8) $$\bar{\alpha }=\left(1-D_{c}\right)\alpha_{A}+\frac{D_{c}}{2}\left(\alpha_{B}+\alpha_{C}\right)$$

where α_A_, α_B_, and *α_C_* are the calculated polarizabilities for Models A, B, and C, respectively. Here, we assumed that the structures corresponding to Models B and C were equally generated in the PI films by crosslinking reactions.

**Figure 4:**
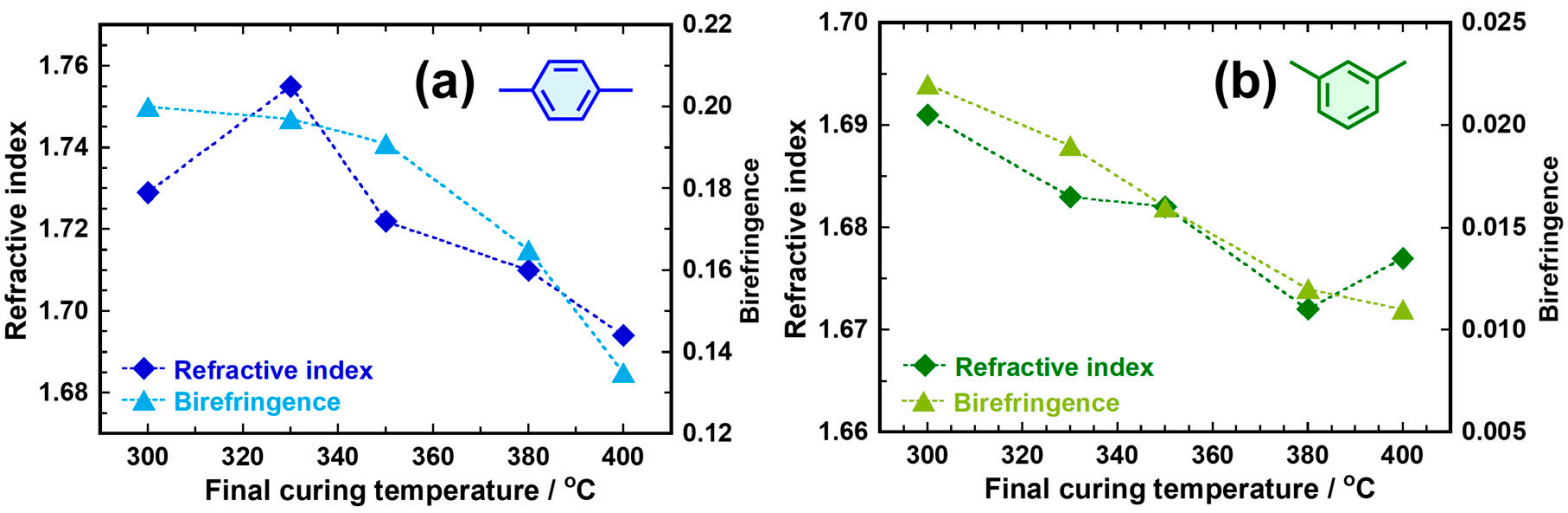
Final curing temperature dependence of average refractive indices (*n*_av_) and in-plane/out-of-plane birefringence (∆*n*) for (**a**) PPD-PIs and (**b**) MPD-PIs.

Figure 4 shows the effect of curing temperature on the average refractive indices (*n*_av_) and birefringence (∆*n*) measured at 1310 nm for the PPD- and MPD-PIs. The PPD-PIs exhibited much larger *n*_av_ and ∆*n* values than those of the MPD-PIs, a result which was attributed to the linear and planar structure of EBPA-PPD (Scheme 1). It should be noted that the ∆*n* of PPD-300 (0.200) was larger than that of *s*BPDA-PPD (0.187), which is one of the most birefringent PIs [18]. In contrast, the ∆*n* of MPD-300 (0.022) was approximately 1/10 of the value of PPD-300, demonstrating the drastic effect of *m*-phenylene linkages, which disrupt the in-plane orientation of PPD-PIs. With an increasing curing temperature, both the *n*_av_ and ∆*n* values gradually decreased, indicating that structures with lower refractive indices and smaller birefringences were generated in the PI films during crosslinking. This view is strongly supported by the significantly smaller calculated refractive indices and intrinsic birefringence of Models B and C compared to those of Model A, as listed in Table S1. As an exception, the sudden increase in *n*_av_ observed for PPD-330 coincides well with the sharp and intense WAXS peaks of the PI, as discussed later. This can be explained by the formation and growth of the ordered structures by curing at 330 °C. In addition, the declined *n*_av_ observed for PPD-350 agreed with the changes in the WAXS pattern.

**Figure 5:**
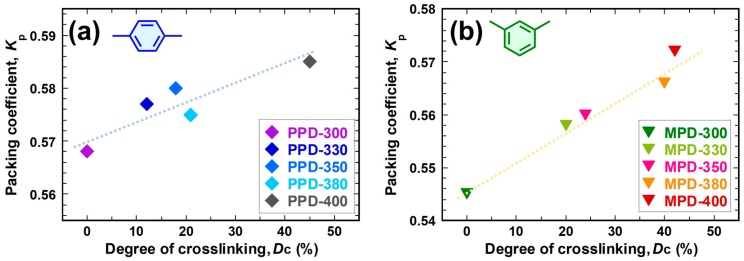
Relation between the degree of crosslinking (*D*_C_) versus molecular packing coefficients; *K*_p_ for (**a**) PPD-PIs and (**b**) MPD-PIs.

Figure 5 shows the relation between the *D*_c_ and *K*_p_ values for the PPD- and MPD-PIs. It should be noted that the EBPA-PI films exhibited larger *K*_p_ values with increasing final curing temperature, which is contrary to the result obtained with the PIs having cross-linkable structures at their termini [10]. This clearly indicates that the dense molecular packing of PI chains occurred upon thermal crosslinking of diphenylethynylene incorporated into the main chain at higher temperatures, which occurred regardless of the isomeric (*p*- or *m*-phenylene) diamine structures.

**Figure 6:**
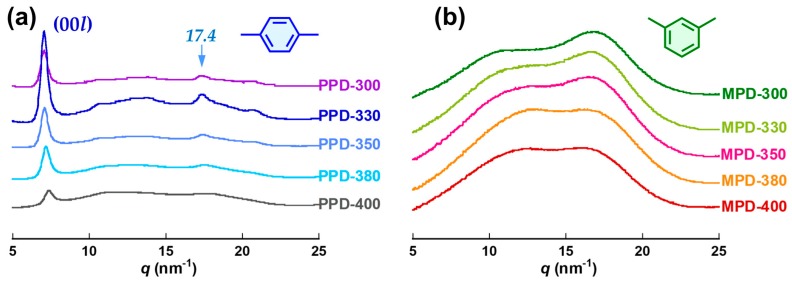
Wide-angle X-ray scattering (WAXS) patterns of the PI films derived from (**a**) PPD and (**b**) MPD.

Figure 6 shows the WAXS patterns of the PPD-PI and MPD-PI films. As seen in Figure 6a, the PPD-330 film exhibited a sharper WAXS peak at 7.05 nm^−1^, which was assigned to the (00*l*) diffraction, without changes in the *q* value compared to the pristine (PPD-300) and other PPD films. In addition, a relatively broad peak at 17.4 nm^−1^, which was assigned to the (*h*00) or (*hk*0) diffraction [19], became more apparent, and the (00*l*) diffraction intensity significantly increased at 330 °C. This clearly indicates that thermal curing at 330 °C promoted not only crosslinking but also the formation of ordered structures. The relatively broad WAXS peaks of PPD-350, and its declined *n*_av_ compared to those of PPD-330, indicate that the ordered structure was partly destroyed by the crosslinking. In general, curing at higher temperatures causes dense chain packing and promotes the formation of ordered structures, leading to increased *K*_p_. It is reasonable that the dense aggregation structure of PPD-330 was formed under appropriate imidization and thermal crosslinking conditions. From the variation of |*P*_200_| values, the in-plane orientation of the main chains in the PI films was observed to be gradually disrupted with increasing final curing temperature. As described above, this indicates that curing at 330 °C promoted the formation of ordered structures accompanied by the reorientation of PI chains, whereas curing at ˃330 °C accelerates the reorientation of the PI chains in the ordered structures during thermo-crosslinking. In contrast, the WAXS patterns of the MPD-PI films, as shown in Figure 6b, showed only broad scatterings without any apparent peaks, indicating that the amorphous nature formed after imidization was maintained even after thermal-crosslinking at ˃330 °C. The gradual displacement of the broad peak at approximately 10 nm^−1^ to larger *q* values reflects the interchain distance shortening caused by the crosslinking reactions [20].

### 3.3. Effects of Crosslinking on the Coefficient of Volumetric Thermal Expansion

**Figure 7:**
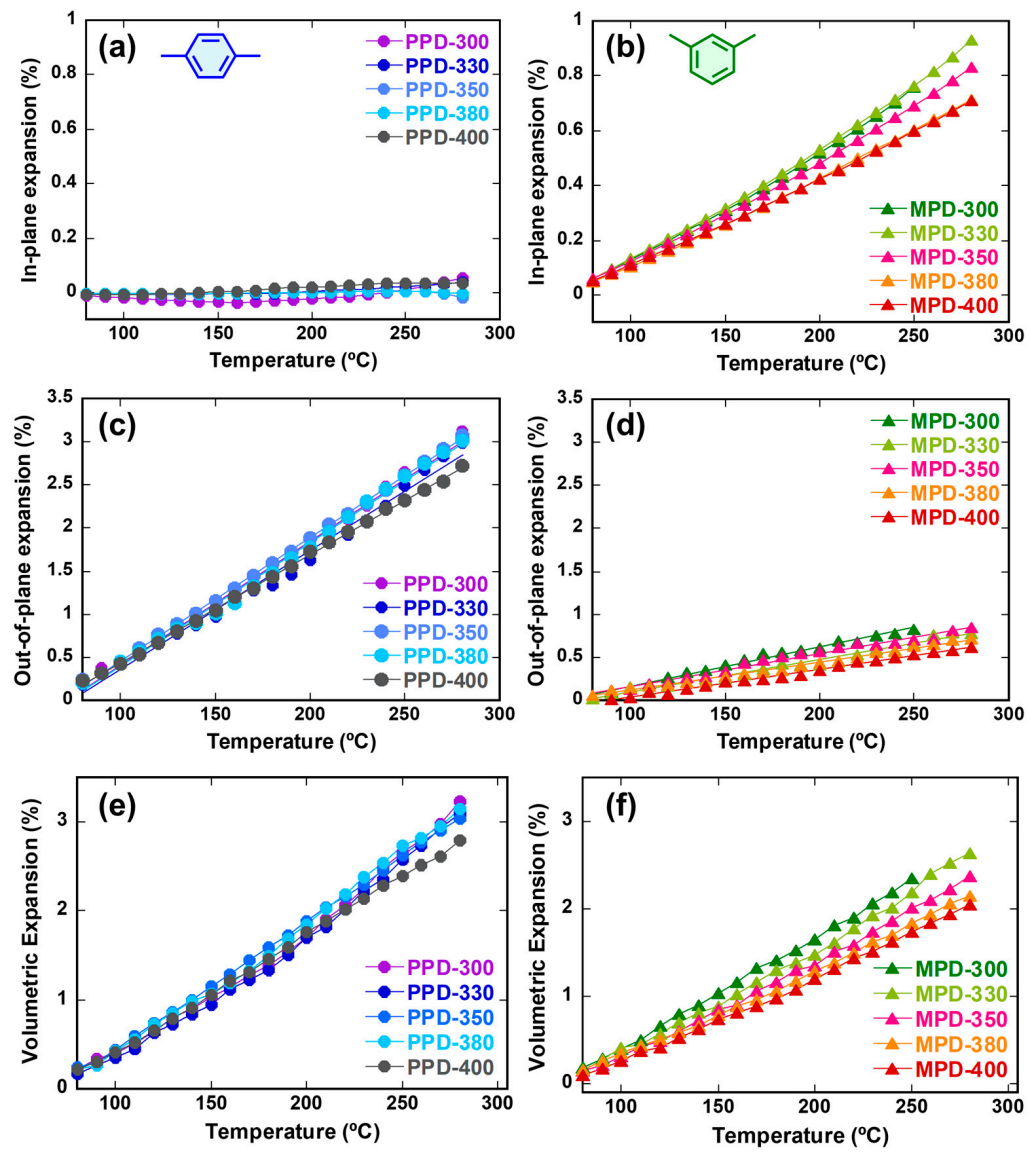
(**a**,**b**) In-plane, (**c**,**d**) out-of-plane, and (**e**,**f**) volumetric thermal expansion behaviors of PI films derived from PPD (left-hand-side) and MPD (right-hand-side).

The in-plane and out-of-plane thermal expansion behaviors of the prepared samples are shown in Figure 7a–d, and the VTE behaviors of the PI films are shown in Figure 7e,f. Based on their VTE curves, the CTE and CVE values were estimated in the temperature range of 80–280 °C, and are listed in Table 4. For the PIs exhibiting lower *T*_g_s of ˂280 °C, the values were estimated in the temperature range of 80 to *T*_g_ − 30 °C, in order to eliminate the influence of *T*_g_. It should be noted that all PPD-PI films exhibited very small CTE_//_ values (0−3 ppm/K) because of their quasi-linear structure, as shown in Scheme 1. The CTE_//_ of the *s*BPDA/PPD PI, with a similar structure to EBDA-PPD, was determined to be 3.1 ppm/K [5], and nearly zero or negative CTE_//_ values were reported for fully aromatic PIs with rigid-rod structures [4,5,21,22,23]. On the contrary, the CTE_⊥_ values obtained in this study were extraordinarily large (144−150 ppm/K), and were slightly reduced by curing at 400 °C (132 ppm/K). In contrast, the MPD-PI films exhibited similar values of CTE_//_ and CTE_⊥_ (32−46 ppm/K), which coincides with their small ∆*n* and |*P*_200_| values, indicating their highly isotropic character. We recently reported that substitution of *m*-phenylene for *p*-phenylene linkages in the PI main chains effectively reduced their CVEs [5]. This is because the local molecular motion of the former PIs are more restricted in the solid state than the latter PIs, even below their respective *T*_g_s, because π-flip rotation at the *m*-phenylene linkage is sterically inhibited, while that at the *p*-phenylene linkage is allowed. The main chains of the PPD-PIs could have undergone both π-flip rotational motion and fluctuation of the PI backbone, whereas MPD-PIs underwent only PI main chain fluctuation. It has been demonstrated that dense PI chain packing, higher *β*-relaxation temperatures, and reduced rotational motions around the phenylene linkage at the secondary relaxation temperature range are key factors for reducing the CVEs of aromatic PI films [5].

The CVE values determined for the PI films using Equation (4) are listed in Table 4. According to our previous reports [5,6], anisotropy in the thermal expansion, *η*, of PI films can be evaluated by the parameter defined as:

(9) $$\eta =\frac{{\mathrm{CTE}}_{⊥}-{\mathrm{CTE}}_{//}}{\mathrm{CVE}}$$

**Figure 8:**
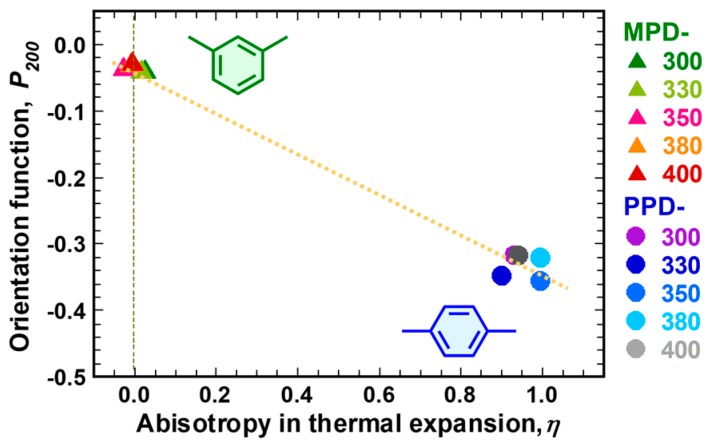
Plots of the orientation function, *P*_200_, as a function of the anisotropic parameter of CTE (*η*).

The calculated *η* values are listed in Table 4 and plotted in Figure 8 against the experimental *P*_200_ values. The MPD-PI films exhibited nearly random orientations (|*P*_200_| ≈ 0) with very small *η* values (−0.044 to +0.042), while PPD-PI exhibited a significantly higher in-plane orientation (|*P*_200_|= 0.222 to 0.317), with very large anisotropy in CTE (+0.994 > *η* > +0.939). The linear relationship shown in Figure 8 clearly indicates that *P*_200_ is the most significant determining factor for η, and the anisotropy in CTE can be controlled by the in-plane orientations of the PI chains. We have previously reported similar relationships [5,6]. Pottiger et al. [24] evaluated the CTE_//_ and CVE values of commercial PI films using TMA measurements and pressure-volume-temperature (PVT) techniques, respectively, where the CTE_⊥_ values were estimated from the CTE_//_ and CVE values. The authors demonstrate that the CTE_//_ value can decrease at the expense of CTE_⊥_ value, because the CVE is nearly intrinsic for each polymer. They also reported that the CVE of PI films does not depend on their thickness (25–125 μm) or chain orientations. Moreover, the present study demonstrates that the CVE of PI films can be reduced by thermal crosslinking.

**Figure 9:**
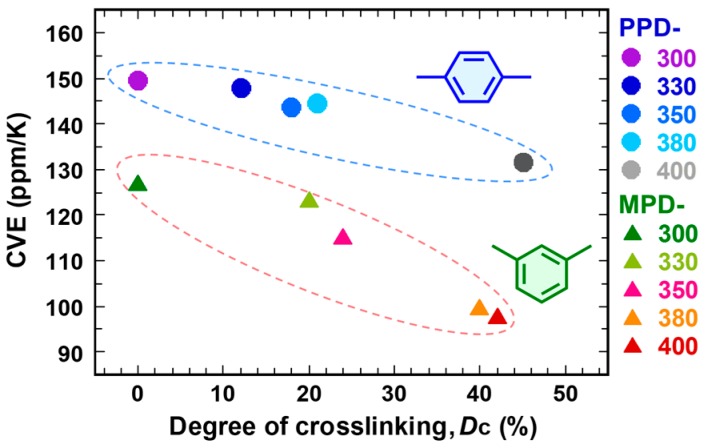
Plots of the CVE values as a function of the degree of crosslinking (*D*_C_) for the PI films.

Figure 9 shows the relationship between CVE and *D*c values measured for the PPD-PI and MPD-PI films. It should be noted that the CVE of the pristine MPD-300 film (127 ppm/K) is much smaller than that of the PPD-300 film (150 ppm/K). As mentioned above, we recently reported that PI films containing bent *m*-phenylene (MPD) linkages exhibited smaller CVEs than those containing linear *p*-phenylene (PPD) linkages [6]. Moreover, both PPD-PIs and MPD-PIs exhibited significant reductions in their CVEs upon thermal crosslinking. The crosslinked PI films cured at 400 °C (PPD-400, MPD-400) exhibited much smaller CVEs than those of the pristine PI films (PPD-300, MPD-300), by −18 and −29 ppm/K, respectively. This clearly demonstrates the effective reduction of the CVE by the incorporation of interchain crosslinking.

**Figure 10:**
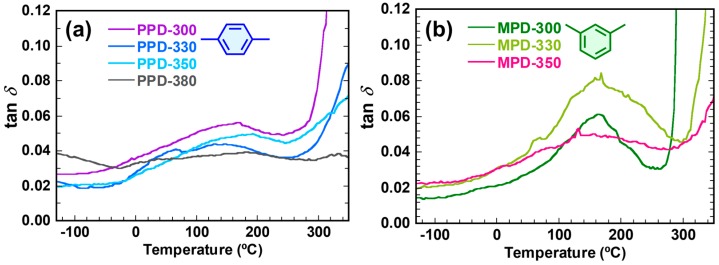
Temperature dependence of tan𝛿 of PI films derived from (**a**) PPD and (**b**) MPD.

To investigate the local motions of the PI chains, their tan δ curves were measured using DMA, and are shown in Figure 10. All PI films exhibited sharp relaxation corresponding to their glass transition (*T*_g_) and broad β-relaxation [6]. The peak temperatures of β-relaxation (*T*_β_) are listed in Table 4. PI films should exhibit relatively dense chain packing with increasing final curing temperatures, owing to the increased degree of crosslinking. The quantity of free volume significantly affected CVE—i.e., if the free volume is the same or decreases after crosslinking, the CVE should be small. The suppression of local molecular motion is important for lowering the CVE. In a previous study, we reported that the PI films exhibiting vigorous local motion below *T*_g_, which is represented by β- and γ-relaxations, demonstrated larger CVE values. Although DMA curves could not be obtained for the PPD-400, MPD-380, and MPD-400 samples because of film brittleness, broad peaks corresponding to β-relaxation were observed for the other PIs between 100 and 250 °C. These relaxation peaks clearly indicate that local molecular motions in the main chain were occurring, i.e., the oscillating motion of the dianhydride moiety or π-flip rotation of the diamine moiety. These DMA curves showed that the intensities of the peaks corresponding to β-relaxation gradually decreased with increasing final curing temperature. This strongly suggests that the local molecular motion relating to VTE was prohibited with an increased degree of thermal crosslinking. Thus, the very small CVE of +98 ppm/K was successfully achieved for the MPD-400 film, which was the smallest CVE among polymers reported to date.

## 4. Conclusions

The *D*_C_ and VTE behaviors of the thermally cross-linkable PI films derived from EBPA dianhydride with diphenylethynylene linkages and isomeric phenylenediamines were systematically investigated using TMA and optical interferometry. The pristine MPD-300 PI film containing *m*-phenylene linkages in the main chain exhibited a much smaller CVE (+127 ppm/K) than its isomeric PPD-300 film containing *p*-phenylene linkages (+150 ppm/K), owing to the suppression of the π-flip motion of the phenyl rings. By analyzing the far-IR spectra of the PI films, the estimated *D*_C_ values increased from 0 to ˃40%, with the final curing temperature increasing to 400 °C. Based on the VTE curves of the PI films, the estimated CVE values were effectively reduced by thermal crosslinking, indicating the potential of crosslinking for reducing the CVE of polymers. Thermal crosslinking of the PIs at higher temperatures (>330 °C) lowered the degree of chain packing by disassembling the polymers’ ordered structure. By analyzing the DMA curves, local molecular motions related to VTE were effectively suppressed in the PI films, with *D*_C_ values of ˃20%. Hence, it can be concluded that intermolecular thermal crosslinking enables the effective reduction of CVE of PI films, and an MPD PI with *m*-phenylene linkages in the main chain crosslinked at 400 °C (MPD-400) exhibited the smallest CVE value (+98 ppm/K) among polymers reported to date.

## Figures and Tables

**Table 1:** Thermal curing conditions of PI films.

Polyimide	Diamine	Post baking	Thermal	Final Curing Condition
			Imidization	
PPD-300	PPD	70 °C ×30 min	300 °C ×1 h	300 °C × 1 h
PPD-330				330 °C × 1 h
PPD-350				350 °C × 1 h
PPD-380				380 °C × 1 h
PPD-400				400 °C × 1 h
MPD-300	MPD			300 °C × 1 h
MPD-330				330 °C × 1 h
MPD-350				350 °C × 1 h
MPD-380				380 °C × 1 h
MPD-400				400 °C × 1 h

**Table 2:** Degree of crosslinking (*D*_C_, %) of the PI films cured at different temperatures.

Polyimide	Final Curing Temperature (°C)				
	300	330	350	380	400
PPD	0	12	18	21	45
MPD	0	20	24	40	42

**Table 3:** Average refractive indices measured at 1310 nm (*n*_av_), in-plane/out-of-plane birefringence (Δ*n*), second-order orientation functions (*P*_200_), and molecular packing coefficients (*K*_p_) of the PI films.

Polyimide	*n*_av_	Δ*n*	*P*_200_	*K*_p_
PPD-300	1.729	0.200	−0.317	0.568
PPD-330	1.755	0.197	−0.313	0.577
PPD-350	1.722	0.191	−0.304	0.580
PPD-380	1.710	0.165	−0.268	0.575
PPD-400	1.694	0.135	−0.222	0.585
MPD-300	1.691	0.022	−0.036	0.545
MPD-330	1.683	0.019	−0.032	0.558
MPD-350	1.682	0.016	−0.027	0.560
MPD-380	1.672	0.012	−0.020	0.566
MPD-400	1.677	0.011	−0.018	0.572

**Table 4:** Coefficient of thermal expansion (CTE) in the in-plane direction of the PI film (CTE_//_) and in the out-of-plane direction (CTE_⊥_), as well as the coefficient of volumetric thermal expansion (CVE), anisotropy in CTE (*η*), and β-transition temperatures determined by the dynamic mechanical analysis (DMA) curves of PI films. The CTEs were estimated in the temperature range of 80 and 280 °C or (*T*_g_ −30 °C).

Polyimide	CTE_//_ (ppm/K)	CTE_⊥_ (ppm/K)	CVE (ppm/K)	*η*	*T*_β_ (°C)
PPD-300	3	144	150	0.944	169 ± 10
PPD-330	2	144	148	0.963	132 ± 10
PPD-350	0	144	144	0.994	192 ± 10
PPD-380	0	144	144	0.994	-
PPD-400	3	126	132	0.939	-
MPD-300	41	46	127	0.042	162 ± 10
MPD-330	43	38	124	−0.044	169 ± 10
MPD-350	38	39	115	0.000	132 ± 10
MPD-380	34	33	100	−0.010	-
MPD-400	33	32	98	−0.005	-

## Supplementary Materials

The following are available online at http://www.mdpi.com/2073-4360/10/7/761/s1, Figure S1: Mid-IR-ATR spectra of (**a**) PPD-PI and (**b**) MPD-PI films cured at different temperatures. Scheme S1: Structures of model compounds used for the DFT calculation to estimate the packing coefficients before and after crosslink reactions. (**a**) Mode A (before crosslinking) and (**b**,**c**) Model B and Model C (after crosslink reactions which form a fused naphthalene or a biphenyl structures). Table S1: Calculated values of average molecular polarizability (α_av_), principal values of polarizability tensor (α_11_, α_22_, α_33_), van der Waals volume (*V*_vdw_), α_av_/*V*_vdw_ values, intrinsic refractive indices parallel and perpendicular to the molecule long axis (*n*^0^_//_, *n*^0^_⊥_), and intrinsic birefringence (Δ*n*^0^) of Models A, B, and C. Figure S2: Calculated far-IR absorption spectra of the model depicted in Figure 3a. Figure S3. Calculated vibration modes of representative far-IR absorption peaks of the model depicted in Figure 3a. Figure S4: Calculated far-IR absorption spectra of model compounds for PPD-PI and MPD-PI. Figure S5: Calculated vibration modes of representative far-IR absorption peaks of the models depicted in Figure S4.
