# Supplementary material for: Effective Reduction of Volumetric Thermal Expansion of Aromatic Polyimide Films by Incorporating Interchain Crosslinking
Source: Polymers (Basel). 2018 Jul 11;10(7):761. doi: 10.3390/polym10070761 (PMC6403670; doi:10.3390/polym10070761)
Supplement: Supplementary file 1 [file polymers-10-00761-s001.pdf]

Supplementary Materials

Effective Reduction of Volumetric Thermal Expansion of Aromatic Polyimide Films by Incorporating Inter-chain Crosslinking

Shinji Ando *, Mari Harada, Tomohiro Okada, and Ryohei Ishige

Department of Chemical Science and Engineering, Tokyo Institute of Technology, Ookayama 2-12-1-E4-5, Meguro-ku, Tokyo, 152-8552, Japan

* Correspondence: sando@polymer.titech.ac.jp; Tel.: +81-3-5734-2137; Fax: +81-3-5734-2889

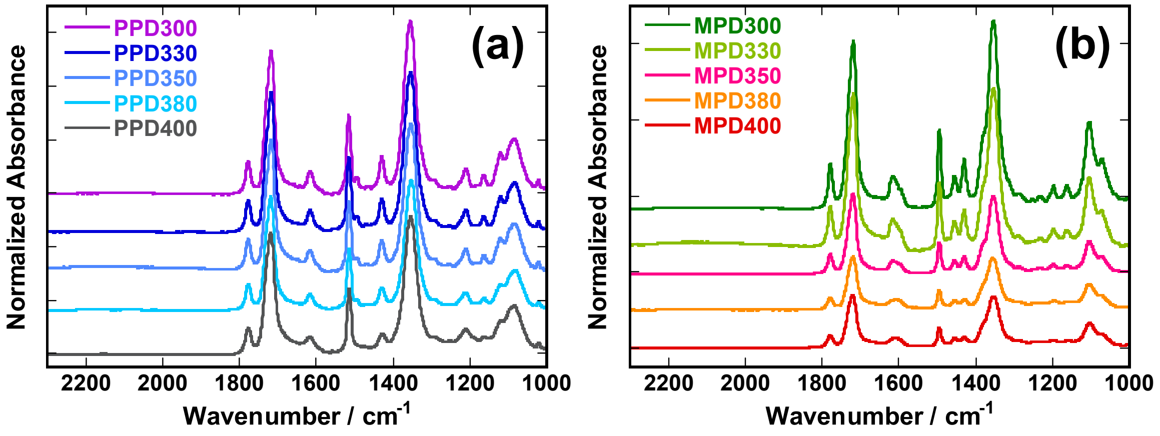

Figure S1. Mid-IR-ATR spectra of (**a**) PPD-PI and (**b**) MPD-PI films cured at different temperatures.

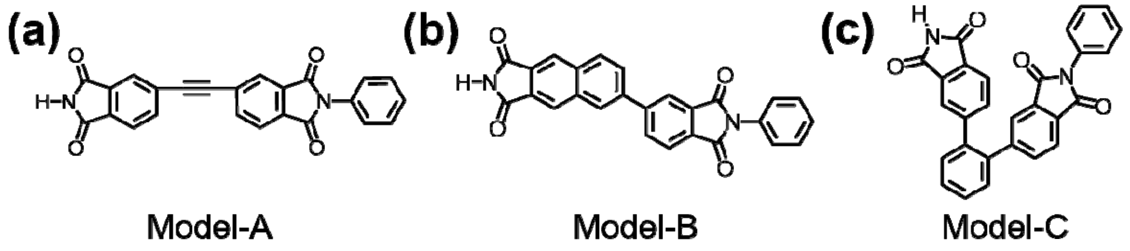

Scheme S1. Structures of model compounds used for the DFT calculation to estimate the packing coefficients before and after crosslink reactions. (**a**) Model-A (before crosslinking) and (**b, c**) Model-B and -C (after crosslink reactions which formed a fused naphthalene or a biphenyl structures).

· Calculation of intrinsic birefringence (Δn^0) using anisotropic molecular polarizability ($\alpha_{//}$ and $\alpha_{\perp}$)

Table S1 shows the molecular polarizabilities (α_{11}, α_{22}, α_{33}), intrinsic refractive indices parallel and perpendicular to the molecule long axis ($n^0_{//}$, $n^0_{\perp}$), the intrinsic birefringence (Δn^0) and van der Waals volume (V_{vdw}) of Model-A, -B, and -C. The values of α_{11}, α_{22}, α_{33} were obtained from the DFT calculations, and those of $n^0_{//}$, $n^0_{\perp}$ were obtained based on the following equations by using experimental average refractive index (n_{av}), in which the n_{av}s of Model-A, -B, and -C were assumed as same as that of PPD-300.

$$n^0_{//} = \left[\frac{\alpha_{//}}{\alpha_{av}}({n_{av}}^2 - 1) + 1\right]^{\frac{1}{2}} \tag{S1}$$

$$n^0_{\perp} = \left[\frac{\alpha_{\perp}}{\alpha_{av}}({n_{av}}^2 - 1) + 1\right]^{\frac{1}{2}} \tag{S2}$$

$$\alpha_{//} = \alpha_{11}, \alpha_{\perp} = \frac{\alpha_{22}+\alpha_{33}}{2}, \alpha_{av} = \frac{\alpha_{11}+\alpha_{22}+\alpha_{33}}{3} \tag{S3}$$

The $\Delta n°$ is given by

$$\Delta n° = n^0_{//} - n^0_{\perp} \tag{S4}$$

Table S1. Calculated values of average molecular polarizability (α_{av}), principal values of polarizability tensor (α_{11}, α_{22}, α_{33}), van der Waals volume (V_{vdw}), α_{av}/V_{vdw} values, intrinsic refractive indices parallel and perpendicular to the molecule long axis ($n^0_{//}$, $n^0_{\perp}$), and intrinsic birefringence (Δn^0) of Model-A, -B, and –C. The calculation procedures were reported elsewhere.[13]

Model	α_{av} (Å^3)	(α_{11}, (Å^3)	α_{22}, (Å^3)	α_{33}) (Å^3)	V_{vdw} (Å^3)	α_{av}/ V_{vdw}	n^0_{av}	$n^0_{//}$	$n^0_{\perp}$	Δn^0
**A**	52.7	90.0	44.6	23.3	330.1	0.160	1.743	2.118	1.515	0.602
**B**	54.5	90.2	47.0	26.3	350.6	0.155	1.715	2.052	1.514	0.539
**C**	53.5	68.2	53.3	39.0	378.8	0.141	1.633	1.767	1.560	0.208

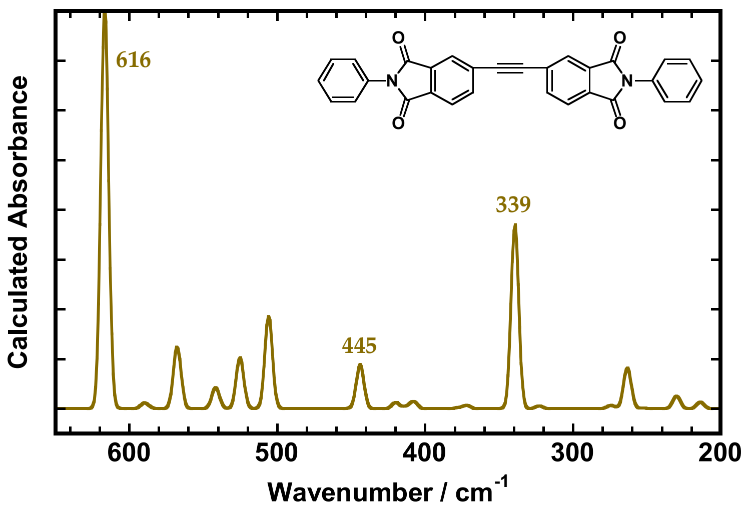

Figure S2. Calculated far-IR absorption spectra of the model depicted in Fig. 3(a).

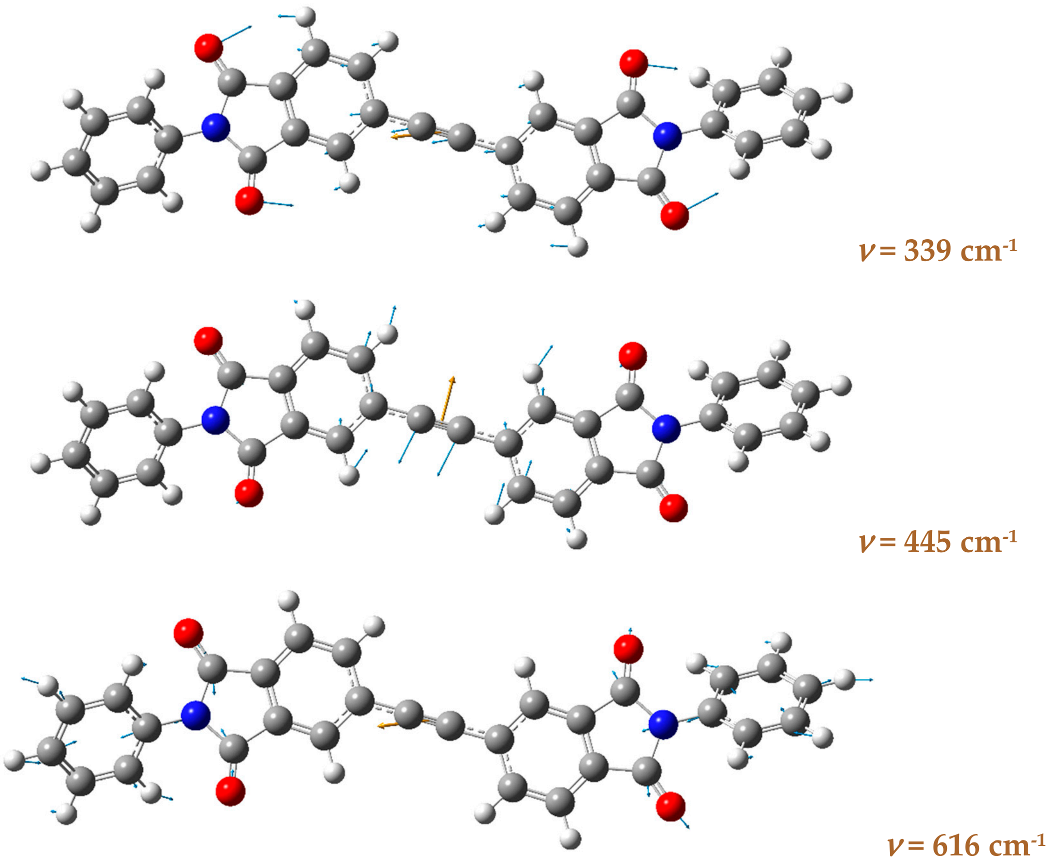

Figure S3. Calculated vibration modes of representative far-IR absorption peaks of the model depicted in Fig. 3(a).

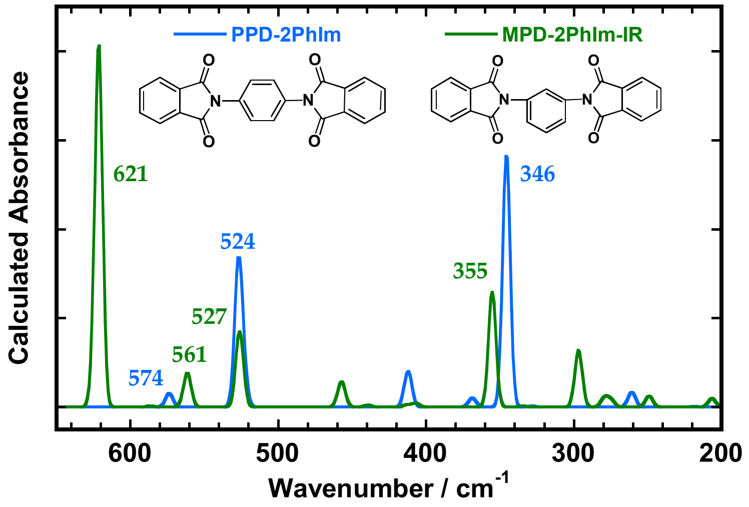

Figure S4. Calculated far-IR absorption spectra of model compounds for PPD-PI and MPD-PI.

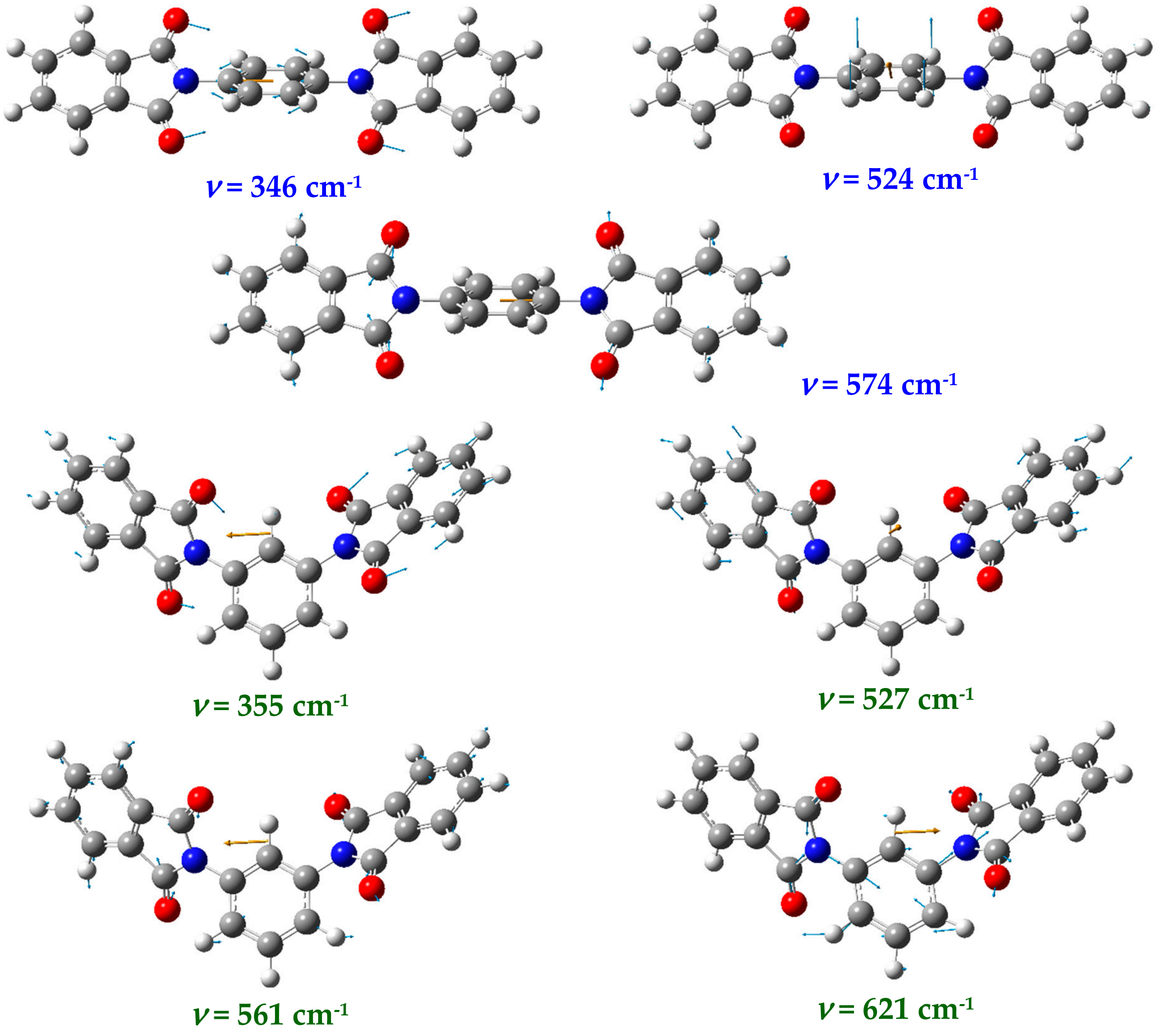

Figure S5. Calculated vibration modes of representative far-IR absorption peaks of the models depicted in Fig. S4.
